# Is Multiple System Atrophy a Prion-like Disorder?

**DOI:** 10.3390/ijms221810093

**Published:** 2021-09-18

**Authors:** Kurt A. Jellinger, Gregor K. Wenning, Nadia Stefanova

**Affiliations:** 1 Institute of Clinical Neurobiology, 1150 Vienna, Austria; 2 Division of Neurobiology, Department of Neurology, Medical University of Innsbruck, 6020 Innsbruck, Austria; gregor.wenning@i-med.ac.at (G.K.W.); Nadia.Stefanova@i-med.ac.at (N.S.)

**Keywords:** multiple system atrophy, α-synuclein, prion-like spreading, neurodegeneration

## Abstract

Multiple system atrophy (MSA) is a rapidly progressive, fatal neurodegenerative disease of uncertain aetiology that belongs to the family of α-synucleinopathies. It clinically presents with parkinsonism, cerebellar, autonomic, and motor impairment in variable combinations. Pathological hallmarks are fibrillary α-synuclein (αSyn)-rich glial cytoplasmic inclusions (GCIs) mainly involving oligodendroglia and to a lesser extent neurons, inducing a multisystem neurodegeneration, glial activation, and widespread demyelinization. The neuronal αSyn pathology of MSA has molecular properties different from Lewy bodies in Parkinson’s disease (PD), both of which could serve as a pool of αSyn (prion) seeds that could initiate and drive the pathogenesis of synucleinopathies. The molecular cascade leading to the “prion-like” transfer of “strains” of aggregated αSyn contributing to the progression of the disease is poorly understood, while some presented evidence that MSA is a prion disease. However, this hypothesis is difficult to reconcile with postmortem analysis of human brains and the fact that MSA-like pathology was induced by intracerebral inoculation of human MSA brain homogenates only in homozygous mutant 53T mice, without production of disease-specific GCIs, or with replication of MSA prions in primary astrocyte cultures from transgenic mice expressing human αSyn. Whereas recent intrastriatal injection of Lewy body-derived or synthetic human αSyn fibrils induced PD-like pathology including neuronal αSyn aggregates in macaques, no such transmission of αSyn pathology in non-human primates by MSA brain lysate has been reported until now. Given the similarities between αSyn and prions, there is a considerable debate whether they should be referred to as “prions”, “prion-like”, “prionoids”, or something else. Here, the findings supporting the proposed nature of αSyn as a prion and its self-propagation through seeding as well as the transmissibility of neurodegenerative disorders are discussed. The proof of disease causation rests on the concordance of scientific evidence, none of which has provided convincing evidence for the classification of MSA as a prion disease or its human transmission until now.

## 1. Introduction

Multiple system atrophy (MSA) is a fatal adult-onset neurodegenerative disorder of uncertain aetiology with a mean incidence of 0.6–0.7 cases per 100,000 person-years. It is clinically featured by various combinations of parkinsonism, cerebellar impairment, autonomic and motor dysfunction due to the degeneration of striatonigral, olivopontocerebellar, and autonomic nervous systems [1,2,3,4] caused by the self-templated misfolding of the protein α-synuclein (αSyn). The core pathological features are fibrillary αSyn-rich glial cytoplasmic inclusions (GCI) mainly involving oligodendroglia [5]. Misfolded αSyn aggregates are also present in Parkinson’s disease (PD) and Lewy body dementia (DLB), which are summarized as α-synucleinopathies [6]. In contrast to PD and DLB, where aggregated αSyn predominantly accumulates within astrocytes and neurons, in MSA, it mainly accumulates within oligodendroglia and to a lesser extent in neurons [7,8,9]. The pathogenic cascade leading to αSyn aggregation and the neurodegeneration of this oligodendroglioneuronal proteinopathy is poorly understood [10,11], but recent studies elucidated the early cellular dysfunction in MSA indicating both increased susceptibility to oxidative stress and disease-related translocation of αSyn to the cell nucleus [12], while others demonstrated mislocalization of myelin-associated oligodendrocyte basic protein (MOBP) and huntingtin protein 1 (HIP1) due to DNA methylation interacting with αSyn in the oligodendrocyte as a pathogenic way of MSA [13]. Converging evidence suggests a “prion-like” spreading of misfolded αSyn “strains” as a pathogenic key event [14,15,16,17,18,19,20,21,22,23,24,25], while others suggested that MSA is a prion disease [26,27,28,29]. The prion hypothesis of human synucleinopathies and the question of whether αSyn is a prion or prion-like are a matter of continuous discussion [15,30,31,32,33,34,35,36,37,38,39,40,41,42]. In both PD and MSA, the debate for and against considering them as prion diseases simply from a prionoid perspective—is ongoing [22,43,44,45,46,47]. This is a critical review of the current data about the prion hypothesis of MSA and other synucleinopathies.

## 2. Self-Propagation of Prionoids

In a series of articles, Prusiner et al. assessed whether αSyn aggregates may act as a prion disease [26,27,29,46,48,49]. A prion was originally described as “a small proteinaceous infectious particle which is resistant to interaction by most procedures that modify nucleic acids” [50]. Later the definition was updated to “proteins that acquire alternative conformations that become self-propagating”, leaving out the requirement for infectivity [51], while, according to others, prions are composed of self-propagating assemblies of misfolded cellular proteins that encode information, generate neurotoxicity, and evolve and adapt in vivo [52]. The prion hypothesis was initially suggested to be incompatible with the observation that the disease agent was capable of inducing multiple disorders in the absence of nucleic acid [53]. This question was resolved with the introduction of the strain hypothesis, which suggests that a disorder is determined by the conversion of the cellular prion protein (PrP^C^) into the pathologic isoform PrP^Sc^ rather than by maturation in a real viral genome [54,55]. In prion diseases, biochemically different species may be responsible for propagation and toxicity. Recent research has shown that in MSA and other neurodegenerative diseases, the protein misfolding is not exclusive to the conversion of PrP^C^ into PrP^Sc^ [56,57]. The recognition that strains have a profound impact across neurodegenerative diseases has provided further insight into their pathogenesis [58,59]. Many observations strengthened the notion that proteins like β-amyloid (Aβ), tau, and αSyn behave like prions. EM structures of recombinant fibrils or patient-derived aggregates containing these proteins show templated misfolding implying that the same mechanisms that induce PrP^Sc^ aggregation also enable the self-templating of these pathologic proteins [56,60]. Similarities are present with the αSyn protein responsible for MSA and other synucleinopathies, and mounting genetic, structural, and biological data support the hypothesis that MSA αSyn prions are distinct from those found in LB diseases, including PD [60]. Carboxy truncations of αSyn promote both its aggregation and toxicity [61]. Specific carboxy truncated forms of αSyn have been detected by immunostaining with antibodies that specifically react with their precise forms showing their specific distribution [41]. In DLB, neuronal inclusions in the SN and amygdala were positive for αSyn cleaved after residues 103, 119, 122, and 125, whereas in MSA GCIs αSyn truncated at residues 103, 115, 119, and 125 were present. In the pontine nuclei, MSA NCIs were reactive to the αSyn x-122 neo-epitope but negative for 103 cleavage. These data demonstrate significant disease-, region-, and cell type-specific differences in carboxy-terminal αSyn in pathological inclusions that may contribute to their distinct strain-like prion properties in the different synucleinopathies, but they also may be related to different prion-like conformational species with various sequences responsible for cleavage [40]. Proteinase K digestion is used to demonstrate the signature of different prion-like protein strains. Altered cleavage profiles due to structural differences are consistent with MSA αSyn profiles structurally different from those in DLB [62]. The differences of the αSyn strains are responsible for the heterogeneity of pathological features and disease processes among synucleinopathies [63] and have been recently proposed to contribute to the more aggressive progression of MSA [64].

Using this modern definition, αSyn aggregates can be classified as prions as they are able to self-multiply during disease, leading to cellular transmission and the spreading of protein aggregates [65,66]. Endogenous αSyn can aggregate through a homotypic (self-seeding) or a heterotypic seeding [67,68], the latter referring to the involvement of other proteins in the initiation of αSyn aggregation [69,70]. The essential point of the prion hypothesis is the notion that self-propagating αSyn aggregates are able to escape from a cell, enter a neighboring one, and then act as a seed to introduce the aggregation of αSyn in the recipient cell [71]. The internalization of the pathogenic conformation of αSyn facilitates its spreading from neuron to neuron [72,73]. αSyn oligomers are internalized primarily through endocytosis [74]. The seeding activity of pathological αSyn species is thought to mediate a process of mutation-dependent aggregation, self-templated propagation of the pathological protein state [32,75]. Intraneuronal αSyn aggregates are triggered by internalized small fibers that do not contact membranes directly, suggesting that this mechanism is relevant to the spreading of aggregated pathologies [76]. Although the molecular mechanisms responsible for spreading pathologic αSyn are poorly understood, a growing body of evidence indicates that de novo misfolding and/or neuronal internalization of aggregated αSyn facilitates conformational templating of endogenous αSyn monomers in a prion-like manner [77]. Recent studies demonstrating that cellular prion protein (PrP^C^) mediates αSyn uptake, localization, and toxicity in vitro and in vivo confirmed previous results which showed that PrP^C^ internalizes soluble misfolded αSyn, indicating its important role in its internalization required for the intercellular spread of αSyn [78]. Cell intrinsic features also may play a critical role in the formation of pathologic αSyn, such as mechanisms that increase endogenous αSyn levels, selective expression profiles in distinct neuron types, altered function of proteins involved in αSyn synthesis and degradation, and oxidative stress. The cell-intrinsic mechanisms that trigger αSyn aggregation and facilitate the conversion of αSyn to a fibrillar pathway to assemble into Lewy bodies, and others, may be important for its self-propagation [79]. Aggregation and propagation in the brain and peripheral organs suggested that αSyn as a prionoid is transmitted from the periphery to the brain via specific pathways [47]. This non-cell-autonomous mechanism was suggested to be similar to that in prion diseases such as Creutzfeldt-Jakob disease, where the prion protein (PrP) in its misfolded form catalyzes the conformational conversion of normal PrP into additional copies of the misfolded PrP (PrP^Sc^) [80]. The ability of prions to self-propagate allows them to spread within host tissues and underlies the transmissible nature of the prion disorders with the capacity to spread between individuals or species [81]. Various proteins have been shown to spread between cells and tissues of the host (for reviews see [82,83,84]), but there is no clear evidence of transmission between individuals, at least by artificial or natural routes [85,86,87]. As the risk of clinical transmission of proteinopathies between humans is critically evaluated, the scenarios for PrP-based prion diseases and their many experimental models must be considered. Not all prion diseases can “spread” horizontally between individuals. Thus, human prion diseases are not contagious in this manner, in contrast to scrapie and others. PrP-based diseases can represent deadly examples of transmissible proteinopathies. However, the ability of an ordered protein assembly to propagate in vitro or in vivo does not necessarily mean that they are transmissible by any casual contacts or medical procedures, or even if they are, that a disease may definitely result from that transmission [86]. There is currently no evidence that αSyn-dependent PD or MSA can be transmitted from person to person [37,85,88]. Many scientists described such intermediate self-propagating protein states as “prion-like”, while others prefer to simply call them all prions [49,89]. The proposed prion-like mechanisms (prionoid) would be restricted to proteins with or among adjacent cells [90], whereas others commented on certain similarities between prion and prionoid and the possibility of prion-like transmissibility of some prionoid strains [91]. Their role in the pathogenesis of neurodegenerative diseases has been critically reviewed [23,92].

**Figure 1:**
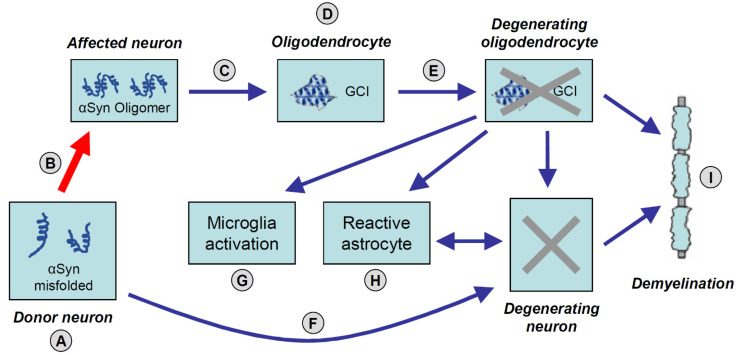
Suggested accumulation and spreading mechanisms of pathological α-synuclein (αSyn) leading to neurodegeneration in MSA. (**A**) Aggregation of αSyn as tetramers and oligomers in neurons. (**B**) “Prion-like” cell-to-cell spreading of abnormally folded αSyn via exosomes (red arrow). (**C**) αSyn aggregates and oligomers released from affected neurons into the extracellular space through exocytosis. (**D**) αSyn aggregation in oligodendroglial cytoplasm. (**E**) Formation of characteristic glial cytoplasmic inclusions (GCIs) in oligodendroglia containing αSyn and p25α. (**F**) Prionoid material can be transmitted directly across synapses causing neurodegeneration. (**G**) Activated micro-/astroglial cells by cytokines released from damaged oligodendrocytes and degenerating neurons. (**H**) Aggregation of αSyn (or other prionoid material) in astrocytes that are also activated by neuronal loss. (**I**) Demyelination caused by damaged oligodendroglia and related to degenerating neurons.

This mechanism would include the misfolding (from α-helix to β-sheet) of native proteins that aggregate into “seeds” that structurally have the capacity to corrupt proteins in their physiological conformation and induce their misfolding. Aggregated αSyn moreover can disrupt glial function, thus contributing to neurodegeneration through various pathways [93]. This process would spread in a chain reaction of misfolding and aggregation ranging from oligomers to large masses of pathologic proteins, leading to neurodegeneration, glial activation, and demyelination [94,95] (Figure 1).

It has been suggested to change the definition of prions to “proteinaceous nucleating particle”, to avoid the infectious implication and to highlight the molecular action of the agents [83]. The term “propagon” has also been suggested to denominate these proteins that act like prions [96]. Both evidence of self-templating propagation in cellular cultures, animal models, and in humans are required to define MSA as a prion disease [97].

## 3. In Vivo and In Vitro Data

The theory that the prion-like mechanisms may underlie neurodegenerative disorders was supported by the demonstration of Lewy pathology after therapeutic transplantation in people with PD, which was discovered not only in the host neurons but also in grafted dopamine neurons about two decades after transplantation. It was hypothesized that prion-like transfer of αSyn might underlie the unexpected pathology in these patients [98,99,100]. Earlier work demonstrating the presence of extracellular αSyn in human plasma and cerebrospinal fluid suggested that αSyn could enter cells from extracellular space [101]. These results as well as many studies using cell culture and animal models gave support to the prion-like hypothesis of the intercellular transfer of αSyn [20,27,102]. In PD brains, αSyn accumulates within neurons and propagates from cell to cell in a prion-like manner [15]. Transmission of pathological αSyn (and tau) between anatomically connected brain regions underlies the stereotypical spread of these and other pathological proteins [59], but it is modulated by the selective vulnerability of cell types and predicted by networks analysis [103]. The spread of αSyn pathology from one cell to another not only within the central nervous system (CNS) and even from one nervous system structure to another in vivo as well as from peripheral locations (such as the gut or olfactory networks into the CNS [47]) has already been convincingly demonstrated [14,20,27,98,104,105,106,107]. Prion-like αSyn propagation has been widely studied in PD [18], where there is evidence of misfolded αSyn in neural cell cultures [108,109]. This spreading pattern resembling that of prions has led to the concept of prion-like propagation of αSyn and tau [52,98,110,111,112], and the Aβ-induced acceleration of tau pathology spreading and its association with prion protein [113,114] (for rev. see [23,39]). This hypothesis in humans is well documented in AD [115], although it is still a hypothesis, like the amyloid cascade [52].

The inoculation of wild-type (WT) mice with synthetic mouse αSyn pre-formed fibrils (PFFs) into striatum induced widespread αSyn pathology and dopaminergic neuronal loss in the substantia nigra compacta, whereas αSyn knockout mice inoculated with PFFs did not develop αSyn pathology [68]. This suggested that endogenous αSyn was necessary to propagate αSyn pathology. Intracerebral injection of sarkosyl-insoluble αSyn homogenates from DLB brain induced αSyn pathology in WT mice [116]. While exogenous human αSyn disappeared a week after inoculation, endogenous mouse αSyn was converted into a pathological form and accumulated in neurons through a prion-like mechanism at three months post-inoculation [117]. Propagation of hyperphosphorylated αSyn occurred along neuronal circuits and involved trans-synaptic transport mechanisms, suggesting that exogenous pathological αSyn can propagate in a neuron-to-neuron manner, but was not associated with motor deficits. A recent review described modeling αSyn propagation with PFF injections and the outcome of these models [118]. The self-propagation of αSyn oligomers in vitro, however, is not sufficient to define them as prions, because they show “seeding” activity rather than infectivity of αSyn [37]. Disease-associated αSyn adopts a conformation that induces it to form oligomers and fibrils with reduced solubility. They become hyperphosphorylated, and contribute to the spatiotemporal spreading of pathology in the CNS, but how the uptake of αSyn varies with the size of oligomers is less clear [74]. The binding of αSyn oligomers to the gap junction protein connexin-32 (Cx32) has been shown to facilitate protein uptake and transfer in neurons and oligodendrocytes [119]. Oligodendroglial p25α is suggested to be responsible for generating a highly pathological αSyn strain in MSA [120]. Oral or intravenous transmission of αSyn fibrils to TgM83+/− mice induces a progressive synucleinopathy associated with overt motor dysfunction and pathological deposition of αSyn aggregates within the brain [121]. Hemizygous TgM83 mice do not develop α-synucleinopathy spontaneously, allowing them to detect disease transmission following extended incubation periods of more than one year post-inoculation. Inoculation studies using homogenates from MSA brain regions lacking detectable αSyn pathology also transmitted neurological lesions to mice indicating that αSyn prion formation precedes neuropathology in the brain, suggesting that the lesions are not limited to affected brain regions [25]. This clearly indicates that αSyn fibrils, like prions, can neuroinvade the CNS after a single oral or intravenous challenge and cause neuropathology. These findings are in agreement with studies demonstrating a cerebral synucleinopathy in transgenic (tg) mice following a peripheral inoculation with αSyn aggregates [122,123,124,125]. Peripheral application of αSyn fibrils that can induce cerebral αSyn pathology suggests that they possess the innate ability to propagate from the periphery to the brain [47,126]. This appears important for understanding the pathogenesis of PD, as it has been speculated that synucleinopathies may originate in the gut before propagating to the brain in a prion-like manner [127].

The intrastriatal injection of PFF αSyn in rodent brains induced a PD-like propagation of Lewy body (LB) pathology together with significant nigrostriatal neurodegeneration. The injection of exogenous αSyn PFFs into the putamen of non-human primates (cynomolgus monkeys) resulted in a significant reduction in dopaminergic neurons in the ipsilateral substantia nigra (−29.3%), downregulation of the dopamine markers tyrosine hydroxylase and Nurr1, associated with LB-like intraneuronal αSyn-positive inclusions; all taken together indicative of early PD [67]. Previous studies using αSyn containing LB-enriched fractions from PD patients into the striatum of macaques showed similar results with the accumulation of αSyn pathology within host neurons and neurodegeneration beginning in dopaminergic terminals, over the course of 12–14 months involving related regions in a PD-like distribution [128]. The injection of synthetic human αSyn fibrils in the striatum of macaque monkeys (Macaca fuscata) showed into the left striatum of Macaca fuscata induced LBs, massive αSyn + NVIs and neurites in the left striatum, some NCIs and neurites in the left SN and bilateral frontal cortex associated with mild neuronal loss and gliosis, while other brain areas were not affected. These results indicated that abnormal αSyn fibrils propagate throughout the brain via projection, association, and commissural fibers, though the progression of αSyn pathology was limited [129]. The intracerebral injection of synthetic αSyn fibrils into adult WT marmoset brains caused abundant αSyn pathologies within only three months post-injection. Robust LB-like inclusions were formed in tyrosine hydroxylase-positive neurons associated with a significant decrease in these neurons suggesting the retrograde spreading of abnormal αSyn and its neurotoxicity [130]. These studies indicate that exogenous αSyn is internalized by dopaminergic terminals, spread to the substantia nigra, and induces PD-like pathology including αSyn aggregation and nigral neuronal degeneration. This supports the ability of abnormal αSyn to propagate to distant brain regions and to trigger neurodegeneration. 

## 4. Prion-Like Properties of αSyn

αSyn has a prion-like property, the propensity to aggregate, that converts its physiological protein conformation into a pathogenic one, forming disease-causing fibrils. The aggregation of these fibrils and subsequent inclusion formations are suggested to interfere with vesicular trafficking and organelle functions in neurons [131]. However, the applicability of the prion hypothesis in α-synucleinopathies remains controversial [45,97]. Although some research groups showed the “infectious” activity of LB extracts from PD brains in mice and also in monkeys [128], demonstrating the prion propensities of αSyn assemblies [44], so far there is no evidence of pathologic αSyn aggregate transmission between individuals leading to the prion-like definition to make a distinction between this protein and actively infectious prions [132]. Several studies demonstrated that pathological αSyn aggregates of GCIs have distinct conformational and biological activities both in vitro and in vivo from those of LBs [26,133,134]. This indicates that αSyn from MSA has a different conformation and a much higher spreading potential than that from PD [133,134]. Injections of MSA brain lysates failed to replicate the oligodendroglial αSyn pathology, raising questions about the pathogenesis of oligodendroglial αSyn aggregates in MSA [72]. WT mice injected with mouse αSyn PFFs developed neuronal αSyn pathology after short post-injection (PI) intervals on the scale of weeks, while oligodendroglial αSyn pathology emerged after longer PI intervals of several months [135]. The protease activity profiles of oligodendrocytes may be distinctive from that of neurons, resulting in the differential αSyn cleavage products responsible for their higher pathogenicity of oligodendroglial αSyn prion-like strains. This would be consistent with a low αSyn expression in oligodendrocytes [136], but that αSyn pathology predominantly spreads in these cells in MSA. It is preferentially propagated in oligodendrocytes despite their lower αSyn expression levels due to a favorable cleavage environment that produces the more potent strains. Aberrant protease activities in MSA could exacerbate this process, but this should be confirmed by future experiments [41]. Both soluble and insoluble fractions of MSA extracts have robust seeding activity, while only the insoluble fraction of PD extracts displayed seeding activity. MSA-seeded inclusions differed from PD-seeded inclusions persisting upon propagation of aggregation to second-generation biosensor cells. It was concluded that PD and MSA feature αSyn conformers with distinct biochemical properties that can be transmitted to αSyn monomers in a cell system. These findings are consistent with the assumption that distinct αSyn strains underlie PD and MSA [137]. The observation that familial Parkinson’s point mutation abolishes MSA prion replication also established that MSA αSyn “prions” are conformationally distinct from the misfolded αSyn in PD [48].

The intracerebral injection of insoluble αSyn into WT mice induced prion-like propagation of phosphorylated αSyn pathology even one month after injection, while injection into αSyn-knockout mice failed to induce any pathology [71]. Abundant oligodendroglial αSyn pathology in white matter developing later was reminiscent of that in MSA. Comparison between young and aged mice injected with mouse αSyn PFFs revealed that PI intervals rather than aging corresponded with oligodendroglia αSyn aggregation in MSA [135]. While these studies indicate that oligodendroglial αSyn pathology can be replicated in WT mice and provide novel insights into the pathological mechanisms of oligodendroglial αSyn aggregations in MSA, the seeded assembly of recombinant human αSyn in vitro did not replicate the structures of αSyn filaments from MSA. This suggests that additional, as yet unknown factors may be essential for the prion-like spreading of αSyn proteinopathies [138]. In MSA, intracerebral inoculation studies in non-human primates, to the best of our knowledge, have not been performed yet.

Homozygous tg mice expressing human αSyn with A53T mutation, termed TgM83+/+ mice, spontaneously develop motor deficits at about one year of age, along with widespread αSyn pathology [139]. Inoculations of young asymptomatic TgM83+/+ mice with brain homogenates from old TgM83+/+ mice induced motor dysfunction [29]. Both intracerebral and peripheral inoculation of brain homogenates from MSA but not from PD patients, were able to produce αSyn pathology in TgM83+/− (hemizygous for the transgene), but not TgM83−/− WT mice, suggesting that different αSyn strains may have different seeding characteristics [27,29,48]. This suggests that αSyn strains different from those observed in PD may be the causative mechanism of MSA [73,137]. It further means that TgM83 mice probably cannot be considered a valid animal model for MSA. This was supported by the fact that Lewy pathology but not MSA-typical GCI pathology was induced by inoculation in αSyn TgM83+/− hemizygous mice, but not in WT mice, suggesting that the A53T αSyn mutation in the SNCA gene plays a critical role in αSyn spreading and self-propagation in this model system. A53T mutation is probably not involved in MSA, but the model is nevertheless responsive to MSA homogenates and, therefore, can be used to study how MSA αSyn adapts, which could be a valid research question, comparable to studies in the prion field where, e.g., hamster prions have been inoculated into mice. Homogenate from diseased MSA patients readily transmits neurodegeneration to TgM83+/− mice [29]. This provided the opportunity to determine if αSyn adopts an alternative confirmation that undergoes self-propagation, which was suggested to become a prion. Nineteen MSA patient samples from three continents transmitted disease to TgM83+/− mice, while those from PD patients did not [26]. Using human embryonic kidney (HEK293T) cells expressing mutated αSyn fused to a yellow fluorescent protein (αSyn140*A53T-YFP). They infected the cells with αSyn isolated from MSA brains but not from PD samples [26]. Studies showing conformational and biological differences between aggregated αSyn species in PD and MSA indicated that the cellular environment affects the aggregation process of αSyn [26,133,134]. The existence of two distinct strains in MSA and PD patients is consistent with the findings that αSyn misfolding into distinct conformations produces differing pathophysiological effects [140,141]. These and other findings suggest that specific strains of αSyn aggregates are responsible for each disease, and may underlie the pathological and clinical diversity of α-synucleinopathies [142,143]. The inoculation of tg mice with different strains of recombinant or brain-derived αSyn aggregates produced clinically and pathologically distinct diseases [56], suggesting that both prion-like spreading and selective vulnerability contribute to the temporal and spatial evolution of αSyn pathology within the brain. Thus, pathogenic αSyn exhibits key hallmarks of prion strains, which provides evidence that disease heterogeneity among the synucleinopathies is caused by distinct αSyn strains [39,63].

The mechanisms of the seeding of αSyn pathology in the nervous system are defined by several factors that can differently influence the pathology among strains, thereby causing distinct disease entities [60,64,144,145]. Therefore, it may be necessary to use disease-specific aggregates in such experiments. In prion disorders, approaches targeting PrP^C^ oligomers are developed based on the observation that only oligomers, not monomers, are infectious [22].

## 5. Multiple System Atrophy: A “Prion” Disease?

There are several challenges to the hypothesis that MSA is a prion-disease. First, endogenous WT αSyn is insufficient to propagate αSyn pathology and mutated αSyn is needed as a template. Tg Nbm mice inoculated with the PFFs of brain homogenates from MSA brains developed “prions”, while the control sample did not. Transmission of αSyn “prions” to a second synucleinopathy model and their ability to propagate between two distinct mouse lines while retaining strain-specific properties were suggested to provide compelling evidence that MSA is a prion disease [28]. Another recent study showed that MSA brain lysates contain sufficient seeding activity to induce αSyn inclusion pathology following neonatal injection in TgM83+/− mice, probably inducing several mechanisms besides conformational templating such as the disruption of normal protein homeostasis and neuroinflammatory reactions. Tg(SNCA*A53T(+/+))Nbm mice developed αSyn pathology in neurons and astrocytes throughout the limbic system, which is in contrast to MSA-inoculated TgM83(+/−) mice, which developed exclusively neuronal αSyn aggregations in the hindbrain that caused motor deficits with advanced disease. In crossover experiments, TgM83(+/−) mice inoculated with mouse-passaged control samples had no effect. The same was seen in that mouse-passaged MSA samples induced αSyn formation in Tg(SNCA*A53T(+/+))Nbm mice, but not in controls. The confirmed transmission of αSyn “prions” to a second synucleinopathy model and the ability to propagate prions between two distinct mouse lines while retaining strain-specific properties were suggested to provide compelling evidence that MSA is a prion disease [28]. However, these and other transmission studies could not explain why in MSA αSyn pathology predominantly accumulates in oligodendroglia as MSA-derived αSyn does not have the ability to induce strain-like cell-specific αSyn aggregation. The intrinsic properties of the A53T αSyn in the TgM83 mouse model have been shown to dominate over any strain features harbored by misfolded αSyn in MSA brains [146]. Previous studies indicated that the “prion-like” progression of synucleinopathy in TgM83 mice depends on the mouse genotype (levels of αSyn expression by the mouse) and type of inoculum [147]. Replication of MSA “prions” in primary astrocyte cultures from TG mice expressing human αSyn showed that human αSyn forms distinct inclusion morphologies and propagates within cultured Tg astrocytes exposed to MSA αSyn. This indicates that αSyn expression dominates the tropism of inclusion formation in certain cells, elucidating the role of astrocytes in the pathogenic mechanism of MSA neurodegeneration [148].

Furthermore, GCIs, the morphological hallmarks of MSA, have never been identified in WT mouse brains inoculated with MSA-derived αSyn [29]. In fact, αSyn aggregates (“prions”) derived from MSA patients generated a neurodegenerative pattern that is atypical for MSA [37]. These, and other findings, indicate that αSyn in MSA may be different from that in PD in “prion-like” properties. The fact that the in vivo phenotype has been observed only from inoculating MSA samples into TgM83+/− mice, but not into WT mice [26] needs further investigation in order to better elucidate this particular issue. However, recent studies have shown that MSA “prions” retain strain specificity after serial propagation in two different Tg(SNCA*A53T) mouse lines. Moreover, the mouse-passaged MSA samples induced αSyn motor deficits despite showing αSyn pathology [68,116]. Tg mice expressing WT αSyn developed αSyn deposition at six months of age but no motor deficits [72]. Second, phosphorylated αSyn aggregates, the morphological hallmarks of MSA, were not detected in oligodendrocytes in MSA-inoculated TgM83+/− mice [26,72]. Third, the intracerebral injection of homogenates from spinal cord tissue from naive motor-impaired TgM83+/+ mice induced robust αSyn pathology that mimics the prion-like pathological changes that occur in TgM83 mice when purified αSyn PFFs are injected to accelerate disease onset, suggesting non-prion-type transmission in A53/αSyn tg mice [149]. Fourth, while αSyn strains may exist, no study has definitely propagated patient-derived seeds from cell-to-cell or mouse-to-mouse, nor fully characterized αSyn strains from MSA vs. PD [137]. Furthermore, they have failed to propagate distinct αSyn conformers in clonal lines as has been shown for tau [150], because aggregate-containing clones lose their aggregation state over time. The variety of seeds, animal models, and methodologies currently prevents clear conclusions regarding αSyn-related spreading and toxicity, as well as translation of preclinical findings to human disease [38]. In recent years, many studies have shown that prion-like proteins share not only the prion replication paradigm but also the specific ability to aggregate in different conformations, i.e., strains, with relevant clinical, pathological, diagnostic, and therapeutic implications, related to the molecular basis of the strain phenomenon in prion and prion-like proteins [39]. To confirm the existence of bona fide αSyn prion strains, it will be necessary to test whether distinct structures propagate through living systems and produce consistent pathology as do tau and PFFs. Further studies will be necessary to define the species that induce αSyn aggregation.

Another problem is the relation between PrP^C^ and αSyn. PrP^C^ knockdown in neuroblastoma cells was shown to attenuate the uptake of recombinant αSyn oligomers with a similar effect observed when comparing αSyn uptake in mouse primary hippocampal neurons, prepared from WT or PrP^C^ knockout mice. The latter developed lower levels of αSyn aggregates in the cortex, striatum, thalamus, and hippocampus, suggesting that PrP^C^ may facilitate the uptake and aggregation of αSyn oligomers [151]. They further showed that the replication of scrapie prions was blocked by αSyn oligomers [152], providing a possible explanation for the observation that Creutzfeldt-Jakob disease patients have a more protracted disease course when there is concomitant synucleinopathy [153]. However, another study found no evidence of binding between PrP^C^ and αSyn oligomers and noted that PrP^C^ neither binds to αSyn oligomers nor mediates their detrimental effects [154]. It could be suggested that there may be different species of αSyn oligomers, which have a different binding capacity with PrP^C^, and it is possible that future studies could demonstrate that both PrP^C^-dependent and -independent pathways could play a role in the pathogenesis of synucleinopathies [155]. Accordingly, aggregated αSyn may be potent in the cross-seeding of prion protein misfolding and aggregation in vitro, producing self-replicating states that can lead to prion diseases upon serial passaging in WT animals [156]. On the other hand, the presence of PrP^Sc^ was required to promote the efficient internalization and spreading of abnormal αSyn between cells. However, recent studies showed that PrP^Sc^ was able to efficiently propagate in the brain of animals even in the absence of αSyn, suggesting that this protein may not act as a key modulator of prion propagation. This suggests that αSyn may take part in this process of self-propagation but is not specifically required for sustaining prion conversion and propagation [157]. Prion strains can interfere with each other, influencing the emergence of a dominant strain, and both environmental and host factors may influence the evolution and distribution of prion strains within a population [158]. Moreover, gene analyses have shown that the homozygous state of positions 129 in the PRNP gene is not a risk factor for MSA and no other variants in the PRNP gene were associated with increased risk for MSA [159]. A review of the clinical histories of patients, who had died of MSA or PD, showed no evidence of neurosurgical transmission [160]. Furthermore, studies of couples whose spouses had autopsy-confirmed PD, PSP, or MSA, did not suggest an increased risk of α-synucleinopathy development in the other spouses [161,162]. Up to the present, there is no evidence of iatrogenic transmission of autopsy-confirmed MSA cases. The current absence of evidence, however, is not evidence of the absence of human transmission of misfolded proteins other than prions and Aβ. In view of the importance of this question, the potential for non-invasive human transmission of proteinopathic neurodegenerative disorders needs further research [163] before any conclusion can be drawn [23].

In conclusion, one may postulate that even if the prion-like spreading of αSyn in experimental model systems may justify the view that the progression of neurodegeneration in MSA reflects the cell-to-cell spread of pathological αSyn, this is not sufficient to define MSA as a classical prion disease. After the injection of αSyn fibrils into the olfactory bulb of WT mice, despite the transneuronal spreading of αSyn aggregates to over 40 other brain regions, even at 18 months PI, there was no loss of mitral cells in the olfactory bulb. The lack of progression of αSyn pathology may be due to the compromise of the neuronal circuitry, and the activation of proteolytic mechanisms in resilient neurons may counterbalance the spread of pathogenic αSyn [164]. This underlines the interpretation that prion-like particles and prions are different entities, and that a more precise definition of both, which is capable of differentiating them from one another, is necessary [37]. Initial work on many prion studies has been hampered by incomplete or atypical transmissions, which were then refined with the advent of better animal models, often as tg mouse models. However, in view of the limited availability of human brain material, it is indispensable to develop new methodologies that enable the production of sufficient amounts of disease-specific aggregates for research in order to enable a deeper understanding of the molecular mechanisms underlying the pathogenesis of MSA—and other synucleinopathies—and to develop novel therapeutic strategies to target αSyn aggregation and disease progression in MSA. Therefore, future work may lead to better transmissions for MSA-causing αSyn conformers once a suitable animal model has been found.

## Data Availability

Data sharing not applicable.

## Abbreviations

Aβ	β-amyloid
αSyn	α-synuclein
CNS	central nervous system
DLB	Lewy body dementia
GCIs	glial cytoplasmic inclusions
LB	Lewy body
LB	Lewy body
MSA	Multiple system atrophy
PD	Parkinson’s disease
PFFs	pre-formed fibrils
PI	post-injection
PrP^C^	cellular prion protein
PrP^Sc^	pathologic PrP isoform
tg	transgenic
WT	wild-type
